# Outcome prediction of cardiac arrest with automatically computed gray-white matter ratio on computed tomography images

**DOI:** 10.1186/s13054-024-04895-2

**Published:** 2024-04-09

**Authors:** Hsinhan Tsai, Chien-Yu Chi, Liang-Wei Wang, Yu-Jen Su, Ya-Fang Chen, Min-Shan Tsai, Chih-Hung Wang, Cheyu Hsu, Chien-Hua Huang, Weichung Wang

**Affiliations:** 1 Department of Computer Science and Information Engineering, National Taiwan University, Taipei, 106216 Taiwan R.O.C.; 2 Department of Emergency Medicine, National Taiwan University Hospital, Taipei, 100225 Taiwan R.O.C.; 3 Department of Medical Imaging, National Taiwan University Hospital, Taipei, 100225 Taiwan R.O.C.; 4 Department of Oncology, National Taiwan University Hospital, Taipei, 100225 Taiwan R.O.C.; 5 Institute of Applied Mathematical Sciences, National Taiwan University, Taipei, 106216 Taiwan R.O.C.

**Keywords:** CT scan, Gray-white matter ratio, Hypoxic-ischemic encephalopathy, Out-of-hospital cardiac arrest, Prognosis, Return of spontaneous circulation, Clinical decision making

## Abstract

****Background**:**

This study aimed to develop an automated method to measure the gray-white matter ratio (GWR) from brain computed tomography (CT) scans of patients with out-of-hospital cardiac arrest (OHCA) and assess its significance in predicting early-stage neurological outcomes.

****Methods**:**

Patients with OHCA who underwent brain CT imaging within 12 h of return of spontaneous circulation were enrolled in this retrospective study. The primary outcome endpoint measure was a favorable neurological outcome, defined as cerebral performance category 1 or 2 at hospital discharge. We proposed an automated method comprising image registration, K-means segmentation, segmentation refinement, and GWR calculation to measure the GWR for each CT scan. The K-means segmentation and segmentation refinement was employed to refine the segmentations within regions of interest (ROIs), consequently enhancing GWR calculation accuracy through more precise segmentations.

****Results**:**

Overall, 443 patients were divided into derivation N=265, 60% and validation N=178, 40% sets, based on age and sex. The ROI Hounsfield unit values derived from the automated method showed a strong correlation with those obtained from the manual method. Regarding outcome prediction, the automated method significantly outperformed the manual method in GWR calculation (AUC 0.79 vs. 0.70) across the entire dataset. The automated method also demonstrated superior performance across sensitivity, specificity, and positive and negative predictive values using the cutoff value determined from the derivation set. Moreover, GWR was an independent predictor of outcomes in logistic regression analysis. Incorporating the GWR with other clinical and resuscitation variables significantly enhanced the performance of prediction models compared to those without the GWR.

****Conclusions**:**

Automated measurement of the GWR from non-contrast brain CT images offers valuable insights for predicting neurological outcomes during the early post-cardiac arrest period.

## Background

Despite considerable resuscitation effort, patients with out-of-hospital cardiac arrest (OHCA) may develop mild to severe hypoxic-ischemic encephalopathy. This condition can lead to variant neurological deficits, including comatose status, imposing significant burdens on family members and physicians, both medically and economically. Hypoxic-ischemic encephalopathy induces brain edema, decreased cortical gray matter attenuation, or loss of normal gray/white differentiation [1]. Hence, early prediction of neurological prognosis is crucial for patients who have experienced cardiac arrest. Several methods for assessing neurological status following resuscitation and the return of spontaneous circulation (ROSC) have been proposed in clinical practice. These include neurological examination, brain non-contrast computed tomography (CT), somatosensory evoked potential (SSEP), serum biomarkers, electroencephalography (EEG), and diffusion-weighted magnetic resonance imaging (DW-MRI) [1–6]. Recent guidelines have proposed brain non-contrast CT as an early prognostic tool to be utilized within 24–72 h after ROSC [6–11]. During the early post-cardiac arrest period, the gray-white matter ratio (GWR) emerges as one of the important indicators of hypoxic-ischemic encephalopathy [12–16]. However, emergency or intensive care physicians cannot perform the quantitative measurement of the GWR using a bedside imaging system. Therefore, measurement should be conducted subjectively and manually at the working station in the radiology department upon request. Nonetheless, an enhanced and objective measurement of the GWR would improve the clinical evaluation and prognostication of hypoxic-ischemic encephalopathy [17, 18].

Hanning et al. [19] proposed an automated, observer-independent probabilistic gray-white matter segmentation algorithm to predict the outcome of 84 patients following cardiac arrest. In 2020, Hannawi et al. [20] developed an automated algorithm to compute the GWR using image registration and atlas segmentation. Similarly, in 2021, Kenda et al. [21] also proposed a comparable method for assessing brain CT scans. However, the accuracy of previous methods significantly relied on precise registration. Although Hannawi et al. [20] addressed artifacts and cerebrospinal fluid (CSF) pulsation by excluding Hounsfield unit (HU) values $\le$ 15, none of the studies adjusted atlas segmentation to account for the difference between the gray and white matter. Furthermore, studies of this nature are rare and most of them were limited to a small dataset. The methodologies proposed in previous studies remain unapplied in clinical practice.

Therefore, this study aimed to predict favorable neurological outcomes by developing an automated method for quantifying the GWR using brain CT scans during the early post-cardiac arrest period, which included image registration and incorporated various pre-processing and post-processing steps. Furthermore, we compared the performance between the automated method and manual methods for measuring the GWR. We also evaluated the effectiveness of incorporating the GWR into a multimodal model to enhance the predictive accuracy during the early post-cardiac arrest period.

## Methods

### Study population and setting

The data were collected retrospectively from the Integrated Medical Database of National Taiwan University Hospital (NTUH-iMD) from January 2009 to December 2019. National Taiwan University Hospital—a tertiary medical center—typically saw approximately 100,000 emergency department visits per year. Eligible patients included the following: (1) adult patients who experienced non-traumatic OHCA, (2) treated in the emergency department, and (3) successfully resuscitated with ROSC. Overall, 544 patients underwent brain CT imaging within 12 h after ROSC and were enrolled in the study. The Institutional Review Board of National Taiwan University Hospital approved this study, along with a waiver of informed consent from the patients’ relatives or physicians, on October 6, 2020 (IRB No. 202004037RINA, Study title: Prognosis and treatment evaluation of post-cardiac arrest patients—a multimodal, autonomic, neuroprognostic model). All procedures performed in this study adhered to the ethical standards set by the responsible committee on human experimentation (institutional or regional) and the Declaration of Helsinki in 1975.

### Patient data acquisition and outcome measurement

All medical history and details of cardiac arrest events were coded in accordance with the Utstein style and extracted from the electronic ambulance and medical records. This included patient characteristics, interventions provided, and outcomes. To predict the outcome in the early post-cardiac arrest period, variables such as age, sex, pre-existing comorbidities, initial rhythm, resuscitation events, non-contrast brain CT findings, hemodynamic parameters, and laboratory results following ROSC were included for further analysis. Brain CT adhered to the post-cardiac arrest care protocol of the medical center during the study period. Briefly, non-contrast brain CT scans were performed when vital signs were relatively stable after ROSC. Patients were sent to the CT examination room after providing informed consent for CT scan studies. The average time to undergo CT after resuscitation was $103\pm 77$ min (median [interquartile range, IQR] = $88~[64-115]$).

The outcome endpoints included favorable neurological outcomes at hospital discharge, which were defined as cerebral performance category (CPC) scores of 1 or 2 and survival to hospital discharge. The CPC score is a validated 5-point scale indicating neurological disability (CPC 1: good cerebral performance; CPC 2: moderate cerebral disability; CPC 3: severe cerebral disability; CPC 4: coma/vegetative state; and CPC 5: brain death). Patients with a CPC score of 1 or 2 generally exhibited adequate cerebral function to live independently. Withdrawal of life sustaining therapy before hospital discharge adhered to the protocol of the medical center. Briefly, at least two attending physicians evaluated the neurological status by examining pupillary light reflexes, spontaneous respiratory drives, and 24-lead electroencephalograms within 7 days after cardiac arrest. Initial brain CT images were not routinely used but could be referenced by physicians during the evaluation process. Decisions regarding the withdrawal of life sustaining therapy were made after discussing with the family of the patients with cardiac arrest in a comatose state.

### Brain CT image analysis and GWR acquisition

#### GWR calculation formulas

This study focused on two GWR calculation formulas: GWR at the basal ganglia level (GWR_b) and a simplified version, GWR_s. GWR_b was determined by summing the HU values of the caudate nuclei (CN) and putamen (PU), then dividing by the sum of HU values of the posterior limb of the internal capsule (PIC) and corpus callosum (CC) in Eq. 1. The simplified GWR (GWR_s) was calculated as the HU value of the PU divided by the HU value of the PIC, as shown in Eq. 2. The simplified GWR was proposed and compared in the study owing to the well-localized characteristics of these two areas in the brain CT images.

1 $$\begin{aligned} {\textrm{GWR}}\_{\textrm{b}}&= \frac{{\textrm{CN}}+{\textrm{PU}}}{{\textrm{CC}}+{\textrm{PIC}}} \end{aligned}$$

2 $$\begin{aligned} {\textrm{GWR}}\_{\textrm{s}}&= \frac{{\textrm{PU}}}{{\textrm{PIC}}} \end{aligned}$$

#### Manual method

**Fig. 1:**
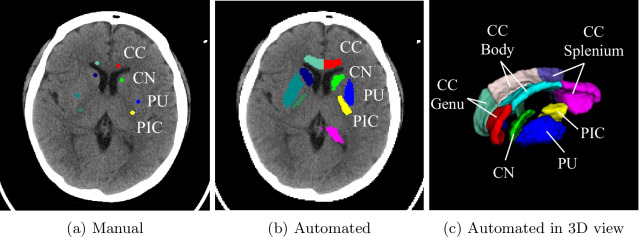
Visualization of **a** manual segmentations, **b** automated segmentations, and **c** automated segmentations in 3D view. CC, corpus callosum; CN, caudate nuclei; PU, putamen; PIC, posterior limb of the internal capsule

The GWR was manually measured to compare its efficacy in predicting outcomes with that of the automatically computed GWR. Following the methodologies of previous studies [22–24], eight regions of interest (ROIs) at the basal ganglia level, including the CC, CN, PU, and PIC, were selected for annotation, (Fig. 1a). Two emergency or critical care physicians who were blinded to the outcomes annotated each circular region. The process involved one doctor providing their assessment initially, and the other doctor subsequently reviewing and verifying the findings. If disagreement persists, a third physician, also blinded to the outcomes, would make the final decision. The area for each circular region was approximately 10 $\mathrm{mm^2}$. The HU for each circular region was calculated by averaging all pixels within the region. The physicians were blinded to the clinical data, survival, and neurological outcomes of the patients before and during the manual annotation of brain CT images.

#### Automated method

**Fig. 2:**
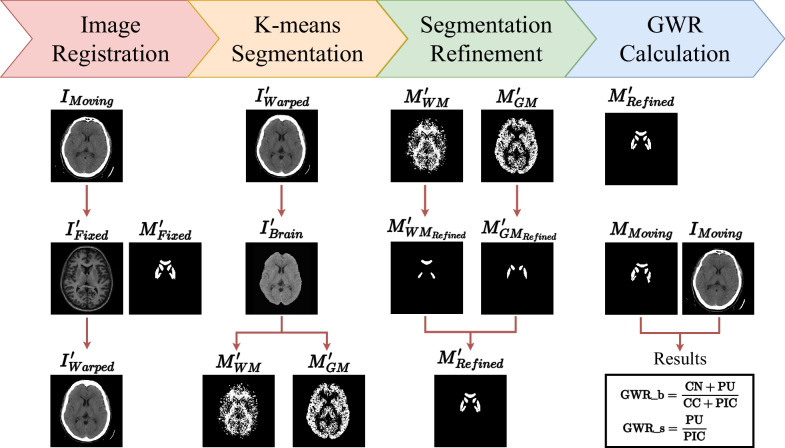
Workflow of the automated method

An automated method was developed to calculate the GWR of head CT scans. Our method comprised four steps: image registration, K-means segmentation, segmentation refinement, and GWR calculation (Fig. 2). For image registration, a nonlinear algorithm from ANTsPy (Version 0.2.9) aligned the CT scan ($I_{Moving}$) with the Eve template ($I'_{Fixed}$) [25, 26]. The objective was to overlay $M'_{Fixed}$ onto the registered CT ($I'_{Warped}$) and subsequently derive all ROI segmentations. However, registration accuracy could be influenced by various factors, potentially affecting the precision of ROI segmentations. Subsequently, K-means segmentation and ROI segmentation refinement were implemented to adjust the $M'_{Fixed}$. First, we extracted the brain from $I'_{Warped}$ and then utilized K-means clustering to obtain the gray ($M'_{GM}$) and the white matter mask ($M'_{WM}$). Following that, techniques including filtering, closing, and opening were employed to refine $M'_{WM}$ and $M'_{GM}$, resulting in the creation of the refined Eve ROIs mask ($M'_{Refined}$). Finally, the eight ROIs (four on each side) were inversely transformed to $M_{Moving}$, and GWR_b and GWR_s were computed according to Eqs. 1 and 2, respectively. Additional file 1 shows further details about the automated method. Compared to the manual method, the automated approach generated a 3D volume for each ROI (Fig. 1b, c). Conversely, the manual method produced a 2D circular region for each ROI (Fig. 1a). Furthermore, Fig. 1b, c delineates the CC into its genu, body, and splenium parts; however, for GWR calculation, all three parts were combined as the entire CC. The automated method offered an advantage in that GWR was calculated using a fixed algorithm, reducing subjectivity among various physicians and ensuring reproducibility. Additionally, the GWR derived from 3D ROI segmentations includes a greater number of voxels, thus providing a more comprehensive characterization of the ROI.

### Excluded CTs management

**Fig. 3:**
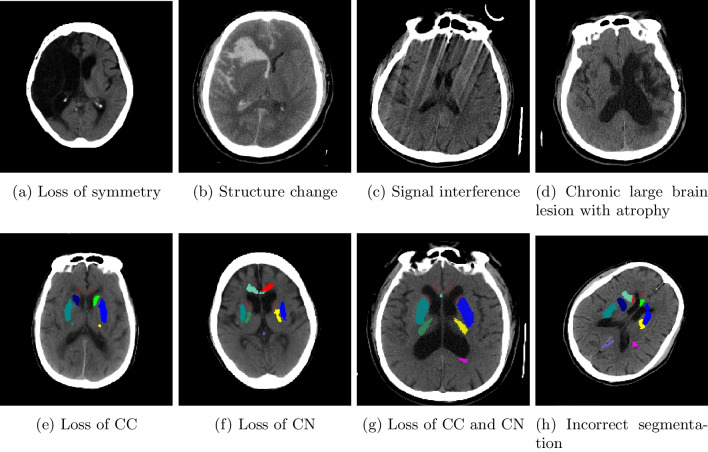
Representative examples of manually (upper row) and automatically (lower row) excluded CTs. In the upper row, manual exclusion criteria were applied, including **a** loss of symmetry, **b** structural change (e.g., intracranial hemorrhage), **c** severe signal interference, and **d** chronic large brain lesion with atrophy. In the lower row, automated exclusion of CTs resulted from inaccurate registration, leading to **e** loss of CC segmentation, **f** loss of CN segmentation, **g** loss of both CC and CN segmentations, and **h** incorrect segmentation. The regions enclosed by dotted lines indicate missing ROI segmentations. CC, corpus callosum; CN, caudate nucleus

The symmetry of brain hemispheres could be influenced by head malpositioning during CT examination or pre-existing localized brain lesions. However, symmetry loss in the automated GWR calculation indicated a lack of symmetry on the brain CT images, potentially leading to GWR miscalculation. If physicians identified a loss of symmetry during the initial evaluation, the case was excluded and defined as a manually excluded CT. The remaining cases were included in the automated calculation. The reasons for manual exclusion included loss of symmetry (N = 2), structural change (including intracranial hemorrhage or brain tumor, N = 11), severe brain atrophy resulting in symmetry loss (N = 4), and marked signal interference or incomplete imaging (N = 5). Figure 3 shows representative examples. After manual exclusion, CTs were further excluded during the automated process by identifying low registration accuracy and any missing ROI segmentations. Subsequently, the automated method was employed for the remaining cases following manual exclusion. However, improper head orientation may cause poor registration with the Eve template, resulting in incorrect segmentation and GWR evaluation (N = 12). Moreover, due to the proximity of the CC and CN to the ventricle, the automated segmentation method may not capture them accurately. In such cases, the CC or CN might be missing, rendering the evaluation of the GWR impossible (N = 5).

### Statistical analysis

Continuous variables, presented as the mean with standard deviation, were compared using Student’s t test, while categorical variables expressed as median with the interquartile range were compared employing the Chi-square test. Pearson’s correlation coefficient (PCC) was utilized to evaluate the correlation between manual and automated calculations of intensities across different brain gray and white matter regions. Receiver operating characteristic (ROC) curves were constructed for manual and automated GWR. The performance of different GWR calculation methods was compared by evaluating the difference in the area under the curve (AUCs) of the ROC curves for each method. Variables with a significance level of $p<0.1$ were included in the multiple logistic regression model. Multiple logistic regression analyses were performed to identify independent variables associated with predicting favorable neurological outcomes. A $p<0.05$ was considered significant. Statistical analyses were performed using R software 4.2.0.

## Results

### Patient enrollment and outcomes

**Fig. 4:**
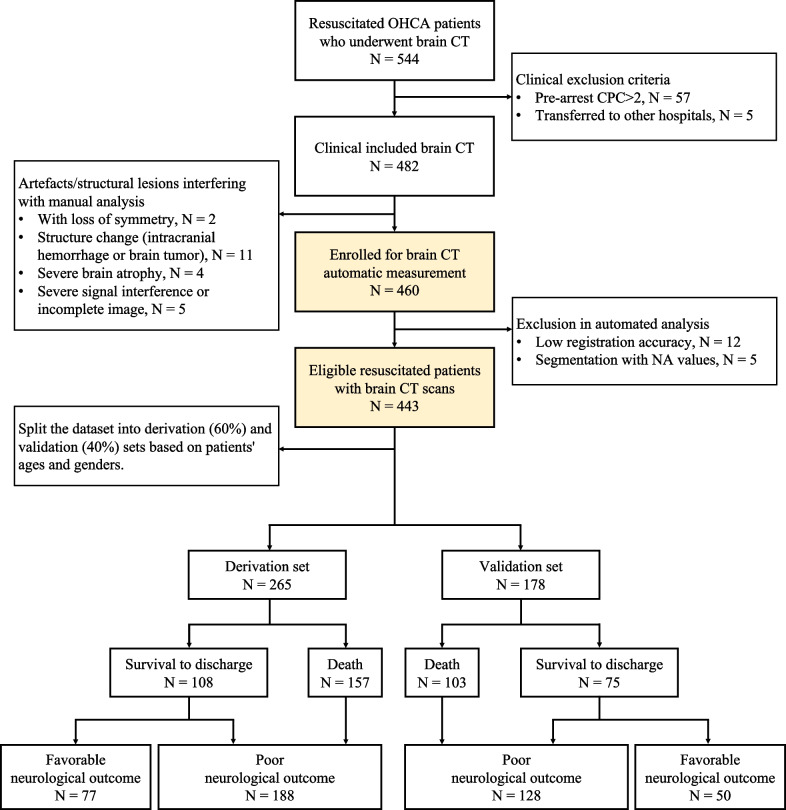
Patient flow chart

Based on the Integrated Medical Database of National Taiwan University Hospital (NTUH-iMD), 544 adult patients underwent successful resuscitation after experiencing non-traumatic OHCA in the emergency department (Fig. 4). Patients with CPC > 2 before cardiac arrest were excluded (N = 57). Additionally, transferred patients were excluded from the analysis due to unknown outcomes (N = 5). After excluding CTs using the manual (N = 22) and automated approaches (N = 17) as previously described, 443 patients with brain CT scans were eligible for further analysis. The dataset was divided into the derivation (60%) and validation (40%) sets based on the age and sex of the patients. In the derivation set, 188 (31 survived and 157 deaths) were in the poor neurological outcome group. In the validation set, 128 (25 survived and 103 died) were in the poor neurological outcome group.

### Baseline characteristics and resuscitation variables of patients

Among the patient characteristics of the entire dataset, lower age was related to survival ($59.2\pm 15.7$ years vs. $66.7\pm 15.4$ years, $p<0.001$) and favorable neurological outcome ($58.0\pm 15.9$ years vs. $65.8\pm 15.5$ years, $p<0.001$). Additionally, a history of malignancy correlated with in-hospital mortality ($4.9\%$ vs. $15.0\%$, $p = 0.001$) and poor neurological outcome ($4.7\%$ vs. $13.3\%$, $p = 0.014$). Survival and favorable neurological outcomes were associated with witnessed collapse, low epinephrine dose, short CPR duration, and higher systolic and diastolic blood pressure Table 1; Additional file 3: Table 1S.

**Table 1:** Baseline characteristics and resuscitation variables of OHCA patients according to neurological outcome

	Neurological outcome								
	Derivation (N = 265)			Validation (N = 178)			Total (N = 443)		
	Good (N = 77)	Poor (N = 188)	P value	Good (N = 50)	Poor (N = 128)	P value	Good (N = 127)	Poor (N = 316)	*P* value
Age (years)	58.0 (15.5)	66.0 (15.2)	$<0.001$	58.0 (16.8)	65.6 (16.0)	0.007	58.0 (15.9)	65.8 (15.5)	$<0.001$
Male	59 (76.6)	127 (67.6)	0.188	38 (76.0)	87 (68.0)	0.384	97 (76.4)	214 (67.7)	0.092
CAD	7 (9.1)	14 (7.4)	0.842	5 (10.0)	11 (8.6)	0.997	12 (9.4)	25 (7.9)	0.735
CVA	2 (2.6)	7 (3.7)	0.932	0 (0)	10 (7.8)	0.094	2 (1.6)	17 (5.4)	0.127
HTN	15 (19.5)	54 (28.7)	0.161	9 (18.0)	31 (24.2)	0.488	24 (18.9)	85 (26.9)	0.100
ESRD	4 (5.2)	17 (9.0)	0.422	4 (8.0)	13 (10.2)	0.876	8 (6.3)	30 (9.5)	0.369
DM	11 (14.3)	36 (19.1)	0.445	5 (10.0)	21 (16.4)	0.395	16 (12.6)	57 (18.0)	0.210
COPD/asthma	1 (1.3)	9 (4.8)	0.318	0 (0.0)	4 (3.1)	0.483	1 (0.8)	13 (4.1)	0.131
Heart failure	2 (2.6)	8 (4.3)	0.773	2 (4.0)	4 (3.1)	1.000	4 (3.1)	12 (3.8)	0.961
Malignancy	2 (2.6)	26 (13.8)	0.013	4 (8.0)	16 (12.5)	0.555	6 (4.7)	42 (13.3)	0.014
*Resuscitation variables*									
Prehospital CPR	65 (84.4)	153 (81.4)	0.682	39 (78.0)	110 (85.9)	0.288	104 (81.9)	263 (83.2)	0.843
Witnessed collapse	65 (84.4)	133 (70.7)	0.030	46 (90.0)	97 (75.8)	0.055	110 (86.6)	230 (72.8)	0.003
Epinephrine (mg)	1.7 (2.3)	4.9 (4.4)	$<0.001$	2.7 (3.9)	4.4 (3.8)	0.012	2.1 (3.1)	4.7 (4.2)	$<0.001$
CPR duration (min)	20.0 (11.1)	23.9 (11.1)	0.009	18.0 (11.4)	23.1 (10.0)	0.008	19.2 (11.2)	23.6 (10.6)	$<0.001$
DBP (mmHg)	86.1 (29.8)	66.6 (24.9)	$<0.001$	80.4 (22.9)	65.5 (22.4)	$<0.001$	83.9 (27.3)	66.1 (23.9)	$<0.001$
SBP (mmHg)	137.6 (47.4)	113.0 (41.0)	$<0.001$	137.0 (41.0)	114.9 (39.4)	0.002	137.4 (44.8)	113.8 (40.3)	$<0.001$
Heart rate (/min)	100.1 (28.8)	102.4 (30.7)	0.549	103.5 (26.7)	101.2 (32.5)	0.625	101.4 (27.9)	101.9 (31.4)	0.865
pH	7.17 (0.15)	7.06 (0.16)	$<0.001$	7.18 (0.14)	7.07 (0.16)	$<0.001$	7.17 (0.15)	7.07 (0.16)	$<0.001$
Lactic acid (mmole/L)	9.0 (4.4)	10.6 (3.6)	0.006	8.8 (4.1)	11.3 (4.8)	0.001	8.9 (4.3)	10.9 (4.1)	$<0.001$
Troponin (ng/L)	4.8 (10.9)	17.2 (77.5)	0.034	15.2 (35.6)	20.3 (86.1)	0.573	8.9 (24.3)	18.5 (81.0)	0.058
*Brain CT image analysis*									
CT timing (min)	106.2 (110.0)	101.1 (50.2)	0.699	85.2 (51.3)	108.3 (93.6)	0.038	97.9 (91.8)	104.0 (71.0)	0.502
Manual_b	1.25 (0.08)	1.19 (0.10)	$<0.001$	1.26 (0.09)	1.20 (0.10)	$<0.001$	1.25 (0.08)	1.19 (0.10)	$<0.001$
Manual_s	1.28 (0.08)	1.20 (0.11)	$<0.001$	1.28 (0.09)	1.21 (0.10)	$<0.001$	1.28 (0.08)	1.21 (0.11)	$<0.001$
Automated_b	1.24 (0.04)	1.18 (0.08)	$<0.001$	1.24 (0.04)	1.19 (0.08)	$<0.001$	1.24 (0.04)	1.19 (0.08)	$<0.001$
Automated_s	1.23 (0.04)	1.17 (0.07)	$<0.001$	1.23 (0.04)	1.17 (0.07)	$<0.001$	1.23 (0.04)	1.17 (0.07)	$<0.001$
*Post-cardiac arrest management*									
PCI	60 (77.9)	49 (26.1)	$<0.001$	33 (66.0)	39 (30.5)	$<0.001$	93 (73.2)	88 (27.8)	$<0.001$
ECMO	15 (19.5)	48 (25.5)	0.373	17 (34.0)	29 (22.7)	0.173	32 (25.2)	77 (24.4)	0.951
TTM	41 (53.2)	82 (43.6)	0.197	18 (36.0)	55 (43.0)	0.497	59 (46.5)	137 (43.4)	0.625

Values are expressed as mean (standard deviation) or n (%) as appropriate

*CAD* coronary artery disease, *CVA* cerebral vascular accident, *HTN* hypertension, *ESRD* end-stage renal disease, *DM* diabetes mellitus, *COPD/Asthma* chronic obstructive pulmonary disease/asthma, *DBP* diastolic blood pressure, *SBP* systolic blood pressure, *PCI* percutaneous coronary intervention, *ECMO* extracorporeal membrane oxygenation, *TTM* targeted temperature management

### Manual and automated measurement of the GWR of brain CT scans

**Fig. 5:**
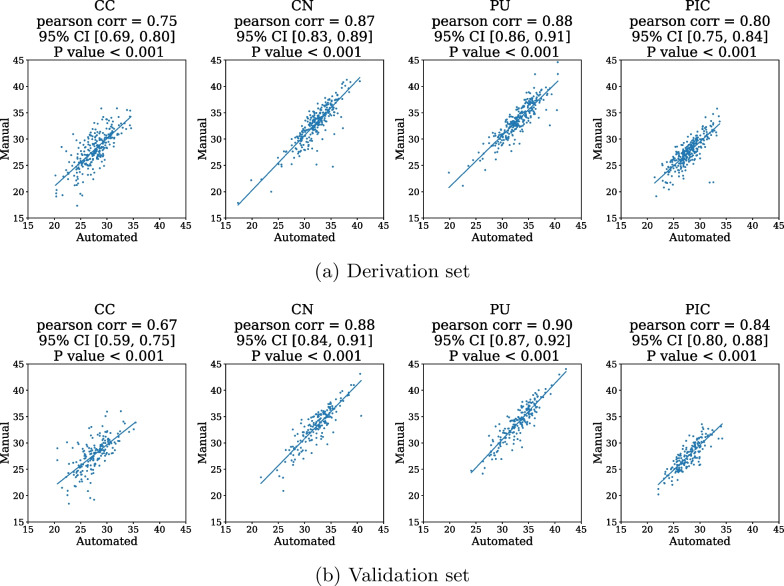
Correlation of HU density between the manual and automated methods in the CN, CC, PU, and PIC in the **a** derivation set and **b** validation set. CC, corpus callosum; CN, caudate nuclei; PU, putamen; PIC, posterior limb of the internal capsule

In the derivation and validation sets, we observed significantly higher GWR values in the favorable neurological outcome group, whether computed manually or automatically (Table 1). This significant difference persisted within the survival-to-hospital discharge group (Additional file 3: Table 1S). To ensure that the HU values of ROI segmentations obtained through the automated method represented the gray and white matter accurately, we evaluated the correlation between the manual and automated methods for four ROI segmentations. Overall, a strong correlation was observed (Fig. 5).

**Fig. 6:**
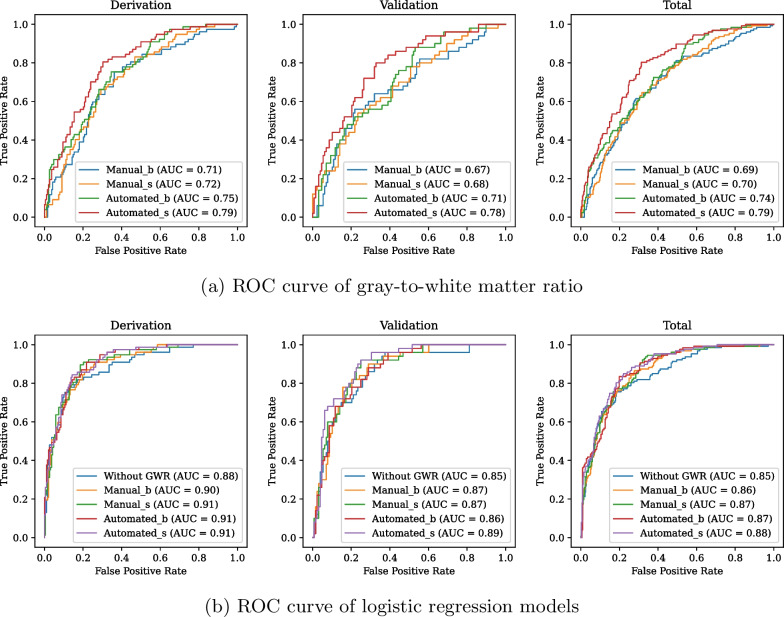
ROC curves and AUCs for predicting favorable neurological outcomes in the **a** GWR and **b** logistic regression models

Subsequently, to assess whether the predictive power of the basal GWR (GWR_b) and simplified GWR (GWR_s) calculated via the automated method was comparable to that of the manual method, we defined the GWR_b calculated through the manual method as Manual_b and the GWR_s as Manual_s. Similarly, the GWR_b calculated via the automated method was defined as Automated_b, and the GWR_s as Automated_s. Figure 6a illustrates the utilization of the GWR in predicting favorable neurological outcomes. Automated_s exhibited the highest AUC values (0.79 and 0.78 in the derivation and validation sets, respectively). The GWR_b and GWR_s showed higher AUCs when calculated through the automated method compared to the manual method. Furthermore, DeLong’s test revealed that Automated_s significantly outperformed Manual_b, Manual_s, and Automated_b with *p* values of 0.001, 0.002, and $<0.001$, respectively (Additional file 3: Table 4S). Table 2 presents the binary classification performance achieved by applying a cutoff value determined based on the Youden index of the ROC curve in the derivation set. Notably, Automated_s exhibited the highest AUC, sensitivity, specificity, positive predictive value (PPV), and negative predictive value (NPV) in the derivation and validation sets. Additional file 2: Fig. 2S shows the scatter plots of manual and automated GWRs versus outcomes.

**Table 2:** Comparison of the performance between the manual method and the automated method in the derivation and validation sets

	AUC (95% C.I.)	Sensitivity (95% C.I.)	Specificity (95% C.I.)	PPV (95% C.I.)	NPV (95% C.I.)	Cutoff value
*Derivation*						
Manual_b	0.71 (0.64–0.78)	0.75 (0.65–0.85)	0.64 (0.57–0.71)	0.46 (0.37–0.55)	0.86 (0.81–0.92)	1.217
Manual_s	0.72 (0.65–0.78)	0.66 (0.55–0.77)	**0.71** (0.64–0.77)	0.48 (0.39–0.58)	0.84 (0.78–0.89)	1.252
Automated_b	0.75 (0.69–0.81)	0.74 (0.64–0.83)	0.65 (0.58–0.72)	0.47 (0.38–0.56)	0.86 (0.80–0.91)	1.217
Automated_s	**0.79** (0.74–0.85)	**0.81** (0.71–0.89)	0.70 (0.63–0.76)	**0.52** (0.43–0.61)	**0.90** (0.85–0.94)	1.204
*Validation*						
Manual_b	0.67 (0.58–0.76)	0.66 (0.52–0.79)	0.54 (0.45–0.62)	0.36 (0.26–0.46)	0.80 (0.71–0.88)	1.217
Manual_s	0.68 (0.60–0.77)	0.62 (0.49–0.75)	0.65 (0.57–0.73)	0.41 (0.30–0.52)	0.81 (0.73–0.89)	1.252
Automated_b	0.71 (0.62–0.79)	0.60 (0.47–0.74)	0.59 (0.51–0.68)	0.37 (0.26–0.47)	0.79 (0.71–0.87)	1.217
Automated_s	**0.78** (0.70–0.85)	**0.78** (0.66–0.89)	**0.67** (0.59–0.75)	**0.48** (0.37–0.59)	**0.89** (0.82–0.95)	1.204

Values in bold demonstrates the best performance of the corresponding metrics in the derivation and validation sets

*C.I.* confidence interval, *PPV* positive predictive value, *NPV* negative predictive value

### GWR as an independent predictor of outcomes

Clinical and resuscitation variables were integrated to evaluate whether the GWR functions serve as an independent factor for favorable neurological outcomes in the early post-cardiac arrest period. Building on previous findings, we established that the simplified GWR might offer superior predictive performance than that of the basal GWR. Multiple logistic regression models were developed with binarized Manual_s and Automated_s using the cutoff values from Table 2. Both Manual_s and Automated_s emerged as independent factors for predicting neurological outcomes (Table 3). Additionally, as demonstrated in Fig. 6b, the models incorporating Manual_b, Manual_s, Automated_b, or Automated_s exhibited higher AUCs than those only clinical variables without the GWR. Moreover, in the total dataset, Manual_s and Automated_s exhibited higher AUCs than Manual_b and Automated_b, respectively, indicating the superior predictive power of the simplified GWR. Further outcomes related to survival to discharge are presented in Additional file 2: Fig. 3S and Additional file 3: Tables 1S–5S. Additional file 3: Table 6S presents the sensitivities, specificities, PPVs, and NPVs for predicting neurological outcomes using manual and automated GWRs at various cutoff values. This table offers valuable information for future meta-analyses. Additional file 3: Tables 7S and 8S showed the AUCs performance at different CT times by hours and median time respectively.

**Table 3:** Multiple logistic regression for predicting favorable neurological outcomes with (a) the manual method and (b) the automated method

	Derivation			Validation			Total		
	Odds ratio	$95\%$ C.I.	*P* value	Odds ratio	$95\%$ C.I.	*P* value	Odds ratio	$95\%$ C.I.	*P* value
(a) With the manual method									
Manual_s $>1.252$	4.13	1.90–8.97	$<0.001$	3.11	1.33–7.22	0.009	3.27	1.94–5.53	$<0.001$
Age	0.98	0.95–1.00	0.107	0.97	0.95–1.00	0.041	0.97	0.96–0.99	0.006
Male	1.43	0.62–3.28	0.398	1.52	0.54–4.31	0.429	1.23	0.68–2.25	0.496
HTN	0.80	0.32–1.97	0.621	1.57	0.52–4.76	0.426	1.03	0.53–1.98	0.940
Malignancy	0.15	0.02–0.86	0.033	1.20	0.29–4.98	0.802	0.38	0.14–1.07	0.068
Witnessed collapse	1.24	0.48–3.17	0.659	2.12	0.63–7.11	0.225	1.35	0.68–2.68	0.396
Epinephrine dose (mg)	0.60	0.48–0.75	$<0.001$	0.86	0.74–1.01	0.064	0.73	0.64–0.82	$<0.001$
CPR duration	1.00	0.97–1.04	0.984	0.95	0.91–0.99	0.023	0.98	0.96–1.01	0.197
DBP	1.04	1.01–1.06	0.005	1.02	0.99–1.06	0.209	1.02	1.01–1.04	0.007
SBP	0.99	0.97–1.00	0.139	1.01	0.99–1.03	0.556	1.00	0.98–1.01	0.410
pH value	14.05	0.77–256.51	0.074	11.51	0.47–283.27	0.135	6.60	0.88–49.64	0.067
Lactic acid	1.00	0.90–1.13	0.941	0.90	0.81–1.01	0.064	0.95	0.88–1.02	0.125
Troponin (ng/L)	0.96	0.93–0.99	0.009	1.00	0.99–1.00	0.502	0.99	0.98–1.00	0.069
ECMO	7.53	2.21–25.69	0.001	4.45	1.48–13.34	0.008	5.23	2.39–11.41	$<0.001$
(b) With the automated method									
Automated_s $>1.204$	5.69	2.57–12.59	$<0.001$	4.81	1.94–11.90	0.001	5.22	2.96–9.19	$<0.001$
Age	0.99	0.96–1.01	0.298	0.97	0.95–1.00	0.062	0.98	0.96–1.00	0.035
Male	1.38	0.59–3.21	0.453	1.61	0.54–4.79	0.396	1.28	0.69–2.38	0.436
HTN	0.74	0.29–1.88	0.521	1.32	0.44–3.94	0.616	1.00	0.51–1.96	0.995
Malignancy	0.10	0.02–0.64	0.015	1.81	0.43–7.60	0.419	0.40	0.14–1.13	0.085
Witnessed collapse	1.67	0.64–4.36	0.294	2.40	0.67–8.63	0.179	1.58	0.78–3.23	0.206
Epinephrine dose (mg)	0.63	0.51–0.78	$<0.001$	0.88	0.75–1.04	0.142	0.75	0.66–0.85	$<0.001$
CPR duration	0.99	0.95–1.03	0.500	0.95	0.91–0.99	0.024	0.97	0.95–1.00	0.073
DBP	1.04	1.01–1.06	0.007	1.02	0.98–1.05	0.374	1.02	1.00–1.04	0.020
SBP	0.98	0.97–1.00	0.064	1.01	0.99–1.03	0.497	0.99	0.98–1.01	0.353
pH value	4.82	0.24–98.17	0.307	5.42	0.21–141.74	0.310	2.96	0.36–24.15	0.310
Lactic acid	0.96	0.85–1.08	0.470	0.91	0.82–1.01	0.081	0.93	0.87–1.00	0.062
Troponin (ng/L)	0.97	0.94–1.00	0.038	1.00	0.99–1.01	0.583	0.99	0.98–1.00	0.075
ECMO	5.12	1.50–17.47	0.009	3.46	1.09–10.96	0.035	3.98	1.78–8.90	$<0.001$

*C.I.* confidence interval, *DBP* diastolic blood pressure, *SBP* systolic blood pressure

## Discussion

The study showed a strong correlation between GWR values obtained through automated measurement and calculation and those derived via manual calculation using the non-contrast brain CT images of patients who had experienced cardiac arrest. The automated GWR exhibited significantly superior predictive power to that of the manually computed GWR. To predict outcomes during the early post-cardiac arrest period, the GWR emerged as an independent predictor in multiple logistic regression models that included clinical and resuscitation variables. Including the GWR in the models led to a significantly higher AUC. The model incorporating the automated GWR achieved the highest AUC value.

Early assessment of prognosis in comatose cardiac arrest survivors remains challenging; however, it remains helpful and crucial for decision making regarding aggressive interventions in post-cardiac arrest care [6–11]. A decreased GWR indicates hypoxic-ischemic encephalopathy and correlates with poor neurological outcomes. However, in a previous study [27], only 12.2% of brain CT scans performed during emergency room stays showed abnormalities as visually diagnosed by a single radiologist. Automated GWR measurement provides essential information more conveniently and efficiently for post-cardiac arrest care. The incorporation of automatically computed GWR enhanced the predictive power for neurological outcomes during the early post-cardiac arrest period as shown in the study. In 2016, Hanning et al. [19] compared automated assessment using normalized probabilistic maps to manual assessment in a small series involving 84 patients. Regarding outcome prediction, the automated GWR (AUC 0.86) demonstrated higher predictive power ($p = 0.021$) than that of the manual GWR (AUC 0.70) with a moderate intra-class correlation coefficient (0.551). However, the clinical endpoint indicating poor CPC following transfer to the general ward may be unreliable, owing to potential late improvement in the study. Conversely, in 2020, Hannawi et al. [20] proposed an automated GWR measurement with a segmentation method based on the JHU-MNI-SS-SS atlas (Eve atlas) [25]. The result showed no significant difference in prognostic performance when comparing models comprising clinical factors only (AUC 0.92) with those comprising clinical factors and the GWR (AUC 0.92). Hannawi et al. [20] registered the head CT to Eve atlas and then derived the segmentation. They also included a post-processing step that discarded voxels with an intensity $\ge$ 100 or $\le$ 15. Their approach appeared to rely heavily on accurate image registration, employing a threshold of 15 for artifact and CSF pulsation removal. However, the segmentation was not adjusted to account for the boundaries between the gray and white matter. For instance, poor registration could result in a portion of the PU overlapping with the white matter. To mitigate potential errors arising from inaccurate image registration, we implemented K-means segmentation and segmentation refinement. However, since the ROIs in CT images were not uniform, the gray and white matter masks derived from the K-means algorithm were fragmented. Direct utilization of these fragmented masks to modify the Eve ROIs mask was not feasible, as the ROIs needed to be complete. Therefore, we used morphology techniques, specifically closing to fill holes within the ROIs and opening to eliminate noise around the ROIs. This process enhanced the accuracy of measuring the CC, CN, PU, and PIC. Additionally, we enrolled a larger number of patients in our study compared to those enrolled in previous automated quantitative GWR studies (Hanning et al. [19]; Hannawi et al. [20]). In our study, a significant correlation was observed between automated and manual GWRs. Additionally, we employed the automated model to measure the 3D volume rather than a small 2D circular area in the manual method. This approach helped in alleviating the variance of HU values within each ROI by averaging a larger number of voxels. In clinical scenarios where manual measurement of the GWR is performed, different physicians with varying levels of experience may select different locations for each ROI, potentially introducing bias to GWR measurements. This variance could account for the lower optimal cutoff value derived by the Youden index for the automated method (1.204 for Automated_s) compared to that of the manual method (1.252 for Manual_s) observed in our study. Although the cutoff values may vary between studies, a value of approximately 1.3 has been suggested as normal, as per the resuscitation guidelines [28]. The cutoff proposed in these guidelines [29, 30] is primarily derived from manual measurement. Similarly, a lower optimal cutoff value of 1.084 was previously identified using an automated quantitative method [19]. In our study, the AUC values for predicting neurological outcomes were significantly higher when using the automated method than with the manual method. This improvement may be attributed to the automated method providing a more representative GWR. However, our method did not provide safe cutoffs for prediction of poor neurological outcome with high specificity on an individual patient basis.

Various studies have reported different AUC values for predicting neurological outcomes using automated GWR, with values of 0.86 [19], 0.73 [20], and 0.79 [21] for brain CT within 24 h after resuscitation. Hence, the timing of obtaining brain CT images may influence prognostic power. As hypoxic-ischemic encephalopathy progresses, the difference between the gray and white matter decreases during the post-cardiac arrest period. The AUCs of the GWR increased from 0.79 for images obtained within 24 h to 0.86 for those obtained after 24 h [21]. GWR changes were more subtle in the earlier post-cardiac arrest period. In a study conducted by Hanning, the median time for obtaining CT images was 8.4 h, while it was 3 h in that conducted by Kenda. In our study, despite a shorter median time of 88 min after resuscitation, the AUC was 0.79. Additionally, we observed an improvement in AUCs from 0.75 obtained within 88 min to 0.83 obtained after 88 min. Automated GWR measurement for predicting neurological outcomes indicates that brain CT in the early post-cardiac arrest period can not only identify potential central nervous system etiologies of cardiac arrest but also provide early prognostic insight for post-cardiac arrest care.

Early prognostication is important in managing patients following cardiac arrest, aiding in decision making regarding invasive procedures and critical care management during the period. Various scoring systems have been proposed for risk stratification; however, the GWR of non-contrast brain CT scans is seldom incorporated into multimodal prognostication. Non-contrast brain CT can be performed immediately after vital signs stabilize after ROSC before transferring patients to the intensive care unit or cardiac catheterization laboratory. The automated measurement of the GWR improves the likelihood of obtaining accurate GWR data after brain CT completion during the early post-cardiac arrest period, eliminating the need for manual measurement. Moreover, specifically incorporating the GWR can significantly improve the predictive power for neurological outcomes when incorporating relevant clinical and resuscitation variables, as revealed in our study. It has been recently suggested that early and delayed brain imaging after ROSC could yield superior predictive power [31]. The automated measurement of the GWR in clinical practice could facilitate decision making during intervention and warrants further investigation.

This study has some limitations. First, this was a retrospective cohort study. Retrospective data collection might include some data loss. Second, inappropriate brain CT images were excluded from the analysis, defining them as cases where the symmetry of brain hemispheres was influenced by head malpositioning during CT examination or prior localized brain lesions, as previously described in the methods section. Cases with severe artifacts or brain disease may cause distortion in the brain structure, potentially leading to inaccuracies in atlas segmentation. Therefore, we excluded these images to ensure accurate sampling of the GWR in the appropriate brain regions. Third, we only considered brain CT scans within 12 h after ROSC for early prognostication during the post-cardiac arrest period. Our timing analysis and previous studies indicate that prognostic accuracy would significantly improve for CTs obtained $>24$ hours after CA. Fourth, the study was conducted at a single-center, specifically a tertiary medical center. The brain CT acquisition system and quality of clinical patient care could vary across different hospitals. However, the automated model utilized for measuring the GWR in the study may be employed in other imaging systems. However, multicenter studies are needed to validate the findings of this study. Fifth, we did not categorize patients who experienced mortality into groups of natural death and withdrawal life sustaining therapy. Although CT scans were not routinely conducted, they were available to treating physicians during the withdrawal of the life sustaining therapy process. The possibility of a self-fulfilling prophecy cannot be excluded. Sixth, the ideal validation cohort should be derived from a prospective cohort study incorporating an adjudication process for neurological outcomes and the timing of CT scans. This limitation arises from its retrospective design. Hence, future studies with a prospective design could provide valuable insights. Finally, performing brain CT scans may introduce bias in retrospective cohort studies. The acquisition of brain CT images was conducted in adherence to the post-cardiac arrest care protocol of the medical center during the study period. While the possibility of not performing brain CT scans after vital signs stabilized was low, it remains a possibility. Hence, future studies employing a prospective design could provide further clarity on these issues.

## Conclusions

Automated measurement with non-contrast brain CT images provides valuable insights into the early post-cardiac arrest period. In this study, we developed an automated method incorporating image registration, K-means segmentation, segmentation refinement, and GWR calculation to determine the GWR. The findings revealed a robust correlation between automated and manual measurements. Furthermore, incorporating the automatically computed GWR can significantly enhance the prediction of favorable neurological outcomes, enabling physicians to gain a comprehensive understanding of the condition of patients for strategic and effective decision making.

### Supplementary information

**Additional file 1: Figure S1.** Automated method workflow.

**Additional file 2: Figure 2S.** The scatter plots of manual and automated GWRs versus (a) neurological outcome and (b) survival to discharge. **Figure 3S.** ROC curves and AUCs for predicting survival to discharge in the (a) GWR and (b) logistic regression models.

**Additional file 3: Table 1S.** Baseline characteristics and resuscitation variables of OHCA patients according to survival to discharge. **Table 2S.** Multiple logistic regression for predicting survival to discharge using (a) the manual method and (b) the automated method. **Table 3S**. Comparison of survival to discharge between the manual method and automated method in the derivation and validation sets.**Table 4S.** DeLong's tests for comparing favorable neurological outcomes and survival to discharge between the manual and automated methods. **Table 5S.** DeLong's tests for comparing favorable neurological outcomes and survival to discharge between multiple logistic regression models with manual and automated GWRs. **Table 6S.** Favorable neurological outcome prediction performance for manual and automated GWRs at various cutoff values. **Table 7S.** Compare AUC performance at different CT timing by hours. Table 8S. Compare AUC performance at different CT timing by median time of performing CT scans in the study.

## Data Availability

The datasets from National Taiwan University Hospital were approved for the current study and are not publicly available. The source codes of this study will be available on GitHub.

## Abbreviations

AUC: Area under the receiver operating characteristic curve
CAD: Coronary artery disease
CAG: Coronary angiography
CVA: Cerebral vascular accident
CC: Corpus callosum
CN: Caudate nuclei
COPD: Chronic obstructive pulmonary disease
CPC: Cerebral performance category
CT: Computed tomography
DBP: Diastolic blood pressure
DM: Diabetes mellitus
DW-MRI: Diffusion-weighted magnetic resonance imaging
ECMO: Extracorporeal membrane oxygenation
EEG: Electroencephalograph
ESRD: End-stage renal disease
GWR: Gray/white matter ratio
HU: Hounsfield unit
HTN: Hypertension
ICU: Intensive care unit
NPV: Negative predictive value
OHCA: Out-of-hospital cardiac arrest
PCC: Pearson’s correlation coefficient
PCI: Percutaneous coronary intervention
PIC: Posterior limb of the internal capsule
PPV: Positive predictive value
PU: Putamen
ROC: Receiver operating characteristic
ROI: Region of interest
ROSC: Return of spontaneous circulation
SBP: Systolic blood pressure
SSEP: Somatosensory evoked potential
TTM: Target temperature management
