# Supplementary material for: Outcome prediction of cardiac arrest with automatically computed gray-white matter ratio on computed tomography images
Source: Crit Care. 2024 Apr 9;28:118. doi: 10.1186/s13054-024-04895-2 (PMC11005205; doi:10.1186/s13054-024-04895-2)
Supplement: Supplementary file 1 — Additional file 1: Figure S1. Automated method workflow. [file 13054_2024_4895_MOESM1_ESM.pdf]

# Illustration of the Automated Method

The proposed automated method comprises four key steps: image registration, K-means segmentation, segmentation refinement, and GWR calculation. In essence, to apply the GWR formulas effectively, we must automatically derive ROI segmentations, including the corpus callosum (CC), caudate nuclei (CN), putamen (PU), and posterior limb of the internal capsule (PIC). The primary concept involves registering the patient's brain CT ($I_{Moving}$) to the Eve template ($I'_{Fixed}$), which contains the ROI mask ($M'_{Fixed}$). Subsequently, we utilize $M'_{Fixed}$ as the ROIs segmentation for the registered CT ($I'_{Warped}$). Finally, we inversely transform $M'_{Fixed}$ from the Eve space to the CT space, obtaining $M_{Moving}$ and allowing us to derive each ROI's HU value and apply the GWR formulas (GWR_b and GWR_s). Note that images or masks in the Eve space are denoted by a "′", and "$I$" and "$M$" represent images and masks, respectively.

However, achieving accurate image registration may be challenging or impossible if the fixed and moving images vary significantly. To address this, we introduce K-means segmentation and segmentation refinement to modify $M'_{Fixed}$, yielding more accurate ROI segmentations. The detailed steps are outlined below:

**Image Registration.** The contrast limited adaptive histogram equalization (CLAHE) algorithm is applied to enhance the local contrast of the brain CT ($I_{Moving}$). Subsequently, it is registered to the Eve template ($I'_{Fixed}$) to obtain the transformation $T(\cdot)$. The enhanced image is utilized for registration only as its intensities have been altered and cannot be applied for GWR calculation.

**K-means Segmentation.** By applying $T(\cdot)$ to the original brain CT ($I_{Moving}$), we obtain $I'_{Warped}$ in the Eve space. The skull is removed through thresholding, erosion, closing, and dilation, resulting in $I'_{Brain}$. Note that HU values of $< 15$ or $> 100$ are discarded during the thresholding operation, as demonstrated in [1]. The K-means clustering algorithm is then employed to separate the brain into gray matter ($M'_{GM}$) and white matter ($M'_{WM}$) masks.

**Segmentation Refinement.** This step is crucial for modifying $M'_{Fixed}$ to derive more accurate ROI segmentations. $M'_{GM}$ and $M'_{WM}$ are restricted inside the ROI by filtering with $M'_{Fixed}$. Closing is applied to fill the gaps among pixels, and opening removes pixels not close enough. Further filtering prevents the PU and PIC from connecting due to their proximity. Finally, $M'_{WM_{Refined}}$ and $M'_{GM_{Refined}}$ are derived and combined into a single mask ($M'_{Refined}$).

**GWR Calculation.** The GWR is calculated using the original brain CT ($I_{Moving}$). Applying the inverse transform $T^{-1}(\cdot)$ to $M'_{Refined}$ yields $M_{Moving}$. Based on $M_{Moving}$ and $I_{Moving}$, the HU values in each ROI are averaged, and GWR formulas (GWR_b and GWR_s) are applied.

However, poor registration can still occur, for example, when the orientation of the CT is improper. In such cases, the ROI mask ($M'_{Fixed}$) cannot be reliably used to locate the ROI on Image ($I'_{Warped}$), resulting in incorrect GWR values. These cases are considered to have low registration accuracy and need to be excluded from the analysis. We quantify the similarity between $I'_{Warped}$ and $I'_{Fixed}$ by evaluating their mutual information [2], which serves as a measure of registration accuracy. Instead of using the entire brain of $I'_{Warped}$ and $I'_{Fixed}$ for evaluation, we focus on the intracranial volume covering CC, CN, PU, and PIC in $I'_{Fixed}$ and the corresponding volume in $I'_{Warped}$. After evaluating the similarities of all 460 CTs, we calculate the mean (M) and the standard deviation (SD) of these 460 similarities. Finally, we exclude cases with a similarity lower than M - 1.96×SD.

## References


[1] Hannawi Y, Muschelli J, Mulder M, Sharrock M, Storm C, Leithner C, et al. Postcardiac arrest neurological prognostication with quantitative regional cerebral densitometry. Resuscitation. 2020;154:101–109.

[2] Mattes D, Haynor DR, Vesselle H, Lewellyn TK, Eubank W. Nonrigid multimodality image registration. In: Medical imaging 2001: image processing. vol. 4322. Spie; 2001. p. 1609–1620.

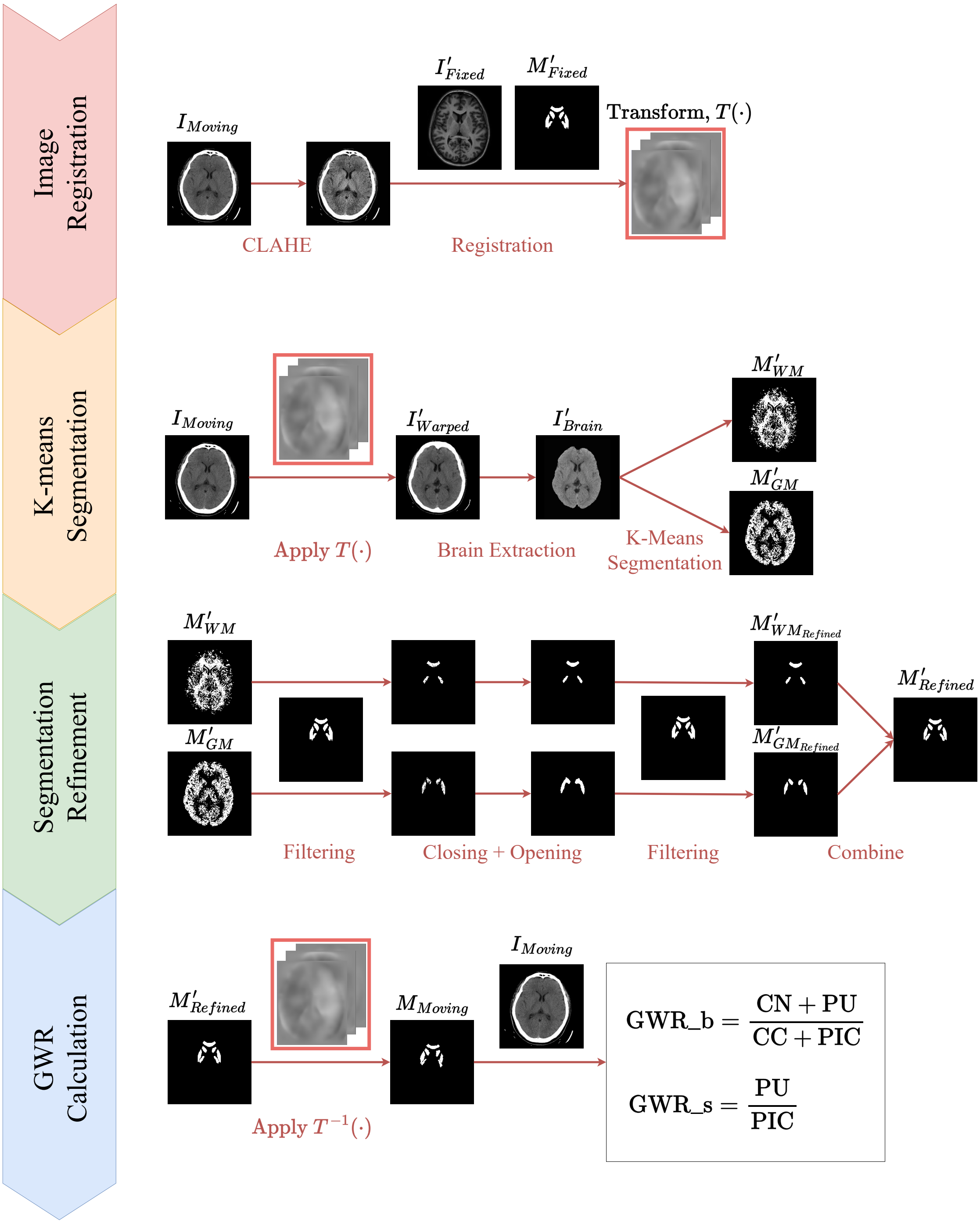


**Supplementary Figure 1S**: Automated method workflow
