# Supplementary material for: Outcome prediction of cardiac arrest with automatically computed gray-white matter ratio on computed tomography images
Source: Crit Care. 2024 Apr 9;28:118. doi: 10.1186/s13054-024-04895-2 (PMC11005205; doi:10.1186/s13054-024-04895-2)
Supplement: Supplementary file 2 — Additional file 2: Figure 2S. The scatter plots of manual and automated GWRs versus (a) neurological outcome and (b) survival to discharge. Figure 3S. ROC curves and AUCs for predicting survival to discharge in the (a) GWR and (b) logistic regression models. [file 13054_2024_4895_MOESM2_ESM.pdf]

Supplementary Figures

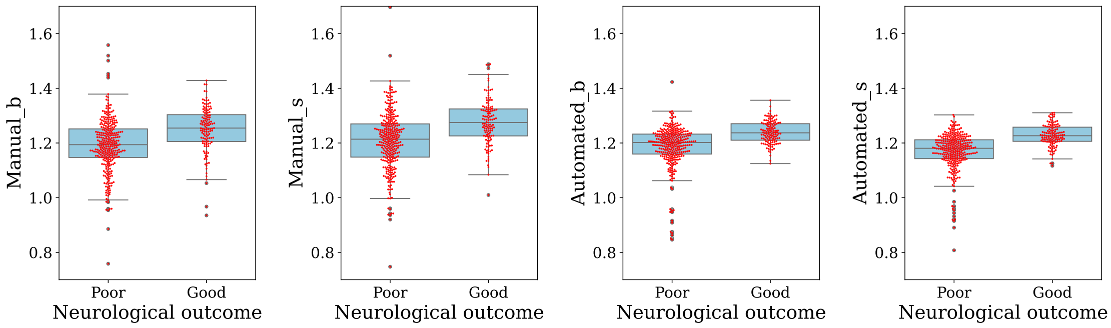

(a) Neurological outcome

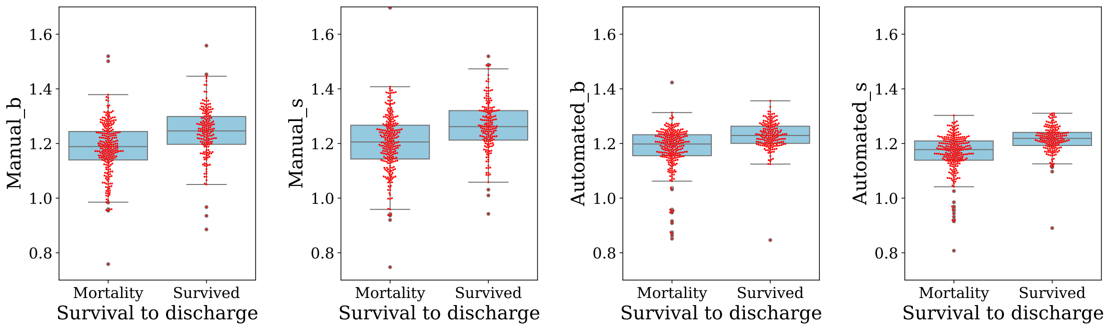

(b) Survival to discharge

Supplementary Figure 2S: The scatter plots of manual and automated GWRs versus (a) neurological outcome and (b) survival to discharge

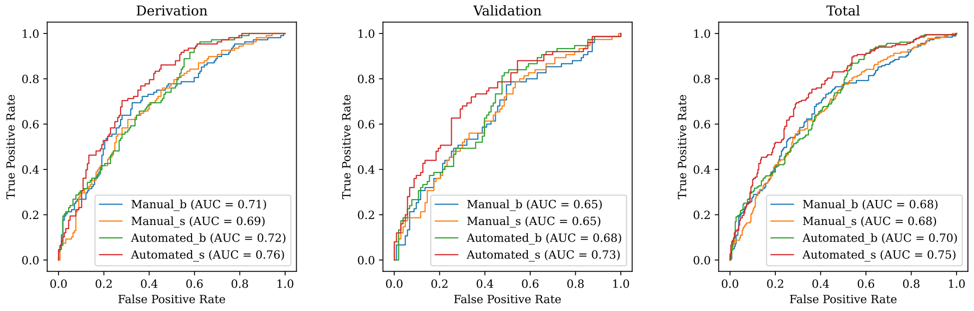

(a) ROC curve of gray-to-white matter ratio

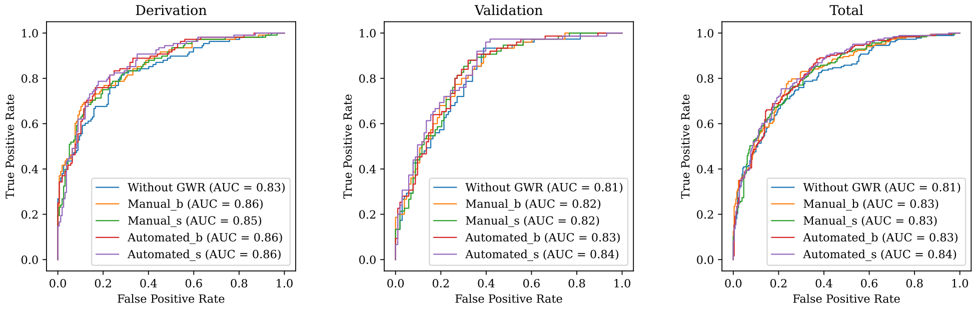

(b) ROC curve of logistic regression models

Supplementary Figure 3S: ROC curves and AUCs for predicting survival to discharge in the (a) GWR and (b) logistic regression models
