# Supplementary material for: Outcome prediction of cardiac arrest with automatically computed gray-white matter ratio on computed tomography images
Source: Crit Care. 2024 Apr 9;28:118. doi: 10.1186/s13054-024-04895-2 (PMC11005205; doi:10.1186/s13054-024-04895-2)
Supplement: Supplementary file 3 — Additional file 3: Table 1S. Baseline characteristics and resuscitation variables of OHCA patients according to survival to discharge. Table 2S. Multiple logistic regression for predicting survival to discharge using (a) the manual method and (b) the automated method. Table 3S. Comparison of survival to discharge between the manual method and automated method in the derivation and validation sets.Table 4S. DeLong's tests for comparing favorable neurological outcomes and survival to discharge between the manual and automated methods. Table 5S. DeLong's tests for comparing favorable neurological outcomes and survival to discharge between multiple logistic regression models with manual and automated GWRs. Table 6S. Favorable neurological outcome prediction performance for manual and automated GWRs at various cutoff values. Table 7S. Compare AUC performance at different CT timing by hours. Table 8S. Compare AUC performance at different CT timing by median time of performing CT scans in the study. [file 13054_2024_4895_MOESM3_ESM.pdf]

Supplementary Tables

1. **Supplementary Table 1S.** Baseline characteristics and resuscitation variables of OHCA patients according to survival to discharge

2. **Supplementary Table 2S.** Multiple logistic regression for predicting survival to discharge using (a) the manual method and (b) the automated method.

3. **Supplementary Table 3S.** Comparison of survival to discharge between the manual method and automated method in the derivation and validation sets.

4. **Supplementary Table 4S.** DeLong's tests for comparing favorable neurological outcomes and survival to discharge between the manual and automated methods.

5. **Supplementary Table 5S.** DeLong's tests for comparing favorable neurological outcomes and survival to discharge between multiple logistic regression models with manual and automated GWRs.
6. **Supplementary Table 6S.** Favorable neurological outcome prediction performance for manual and automated GWRs at various cutoff values.

7. **Supplementary Table 7S.** Compare AUC performance at different CT timing by hours.

8. **Supplementary Table 8S.** Compare AUC performance at different CT timing by median time of performing CT scans in the study.

Supplementary Table 1S: Baseline characteristics and resuscitation variables of OHCA patients according to survival to discharge

	Survival to Discharge								
	Derivation (N=265)			Validation (N=178)			Total (N=443)		
	Survived (N=108)	Mortality (N=157)	P value	Survived (N=75)	Mortality (N=103)	P value	Survived (N=183)	Mortality (N=260)	P value
Age (years)	59.6 (15.4)	66.4 (15.2)	< 0.001	58.6 (16.4)	67.0 (15.8)	0.001	59.2 (15.7)	66.7 (15.4)	< 0.001
Male	82 (75.9)	104 (66.2)	0.045	57 (76.0)	68 (66.0)	0.203	139 (76.0)	172 (66.2)	0.034
CAD	8 (7.4)	13 (8.3)	0.978	5 (6.7)	11 (10.7)	0.510	13 (7.1)	24 (9.2)	0.534
CVA	4 (3.7)	5 (3.2)	1.000	1 (1.3)	9 (8.7)	0.074	5 (2.7)	14 (5.4)	0.263
HTN	23 (21.3)	46 (29.3)	0.188	13 (17.3)	27 (26.2)	0.223	36 (19.7)	73 (28.1)	0.056
ESRD	4 (3.7)	17 (10.8)	0.060	9 (12.0)	8 (7.8)	0.490	13 (7.1)	25 (9.6)	0.449
DM	16 (14.8)	31 (19.7)	0.385	8 (10.7)	18 (17.5)	0.291	24 (13.1)	49 (18.8)	0.141
COPD/Asthma	2 (1.9)	8 (5.1)	0.301	1 (1.3)	3 (2.9)	0.849	3 (1.6)	11 (4.2)	0.208
Heart failure	3 (2.8)	7 (4.5)	0.706	2 (2.7)	4 (3.9)	0.981	5 (2.7)	11 (4.2)	0.566
Malignancy	3 (2.8)	25 (15.9)	0.001	6 (8.0)	14 (13.6)	0.354	9 (4.9)	39 (15.0)	0.001
**Resuscitation variables**									
Prehospital CPR	93 (86.1)	125 (79.6)	0.232	58 (77.3)	91 (88.3)	0.078	151 (82.5)	216 (83.1)	0.979
Witnessed collapse	87 (80.6)	111 (70.7)	0.095	66 (88.0)	76 (73.8)	0.032	153 (83.6)	187 (71.9)	0.006
Epinephrine (mg)	2.3 (3.1)	5.1 (4.4)	< 0.001	2.9 (3.5)	4.7 (4.0)	0.001	2.6 (3.3)	5.0 (4.2)	< 0.001
CPR duration	21.0 (11.1)	24.0 (11.1)	0.035	18.7 (10.6)	23.8 (10.2)	0.002	20.1 (10.9)	23.9 (10.8)	< 0.001
DBP	81.5 (29.0)	65.9 (25.1)	< 0.001	75.6 (25.0)	65.4 (21.4)	0.005	79.1 (27.5)	65.7 (23.7)	< 0.001
SBP	132.9 (46.0)	111.3 (41.0)	< 0.001	130.8 (42.6)	114.0 (38.4)	0.007	132.1 (44.5)	112.4 (40.0)	< 0.001
Heart rate (/min)	99.9 (28.5)	103.0 (31.3)	0.410	99.5 (30.0)	103.5 (31.6)	0.397	99.8 (29.0)	103.2 (31.4)	0.239
pH	7.15 (0.16)	7.06 (0.15)	< 0.001	7.15 (0.14)	7.06 (0.16)	< 0.001	7.15 (0.15)	7.06 (0.16)	< 0.001
Lactic acid (mmole/L)	9.2 (4.4)	10.8 (3.3)	0.001	9.2 (4.1)	11.6 (5.0)	0.001	9.2 (4.3)	11.1 (4.1)	< 0.001
Troponin (ng/L)	7.8 (31.8)	17.6 (81.1)	0.170	12.1 (29.8)	23.8 (95.6)	0.245	9.5 (31.0)	20.1 (87.0)	0.073
**Brain CT image analysis**									
CT timing (min)	102.5 (95.0)	102.6 (52.2)	0.995	96.8 (104.1)	105.4 (66.8)	0.528	100.2 (98.6)	103.7 (58.3)	0.663
Manual_b	1.24 (0.09)	1.18 (0.09)	< 0.001	1.24 (0.10)	1.20 (0.10)	0.001	1.24 (0.09)	1.18 (0.10)	< 0.001
Manual_s	1.26 (0.09)	1.20 (0.11)	< 0.001	1.26 (0.09)	1.21 (0.10)	< 0.001	1.26 (0.09)	1.20 (0.11)	< 0.001
Automated_b	1.23 (0.04)	1.18 (0.08)	< 0.001	1.23 (0.06)	1.19 (0.07)	< 0.001	1.23 (0.05)	1.18 (0.08)	< 0.001
Automated_s	1.22 (0.04)	1.16 (0.08)	< 0.001	1.21 (0.06)	1.17 (0.06)	< 0.001	1.22 (0.05)	1.16 (0.07)	< 0.001
**Post-cardiac arrest management**									
PCI	70 (64.8)	39 (24.8)	< 0.001	45 (60.0)	27 (26.2)	< 0.001	115 (62.8)	66 (25.4)	< 0.001
ECMO	23 (21.3)	40 (25.5)	0.523	21 (28.0)	25 (24.3)	0.698	44 (24.0)	65 (25.0)	0.906
TTM	63 (58.3)	60 (38.2)	0.002	35 (46.7)	38 (36.9)	0.248	98 (53.6)	98 (37.7)	0.001

Values are expressed as mean (standard deviation) or n (%) as appropriate.
Abbreviations: CAD, coronary artery disease; CVA, cerebral vascular accident; HTN, hypertension; ESRD, end-stage renal disease; DM, diabetes mellitus; COPD/Asthma, chronic obstructive pulmonary disease/asthma; DBP, diastolic blood pressure; SBP, systolic blood pressure; PCI, percutaneous coronary intervention; ECMO, extracorporeal membrane oxygenation; TTM, targeted temperature management

Supplementary Table 2S: Multiple logistic regression for predicting survival to discharge using (a) the manual method and (b) the automated method

(a) With the manual method

	Derivation			Validation			Total		
	Odds Ratio	95% C.I.	P value	Odds Ratio	95% C.I.	P value	Odds Ratio	95% C.I.	P value
Manual_s > 1.244	2.36	1.25-4.45	0.008	2.93	1.37-6.26	0.006	2.53	1.60-4.02	< 0.001
Age	0.97	0.95-1.00	0.020	0.96	0.94-0.99	0.004	0.97	0.95-0.99	< 0.001
Male	1.44	0.73-2.86	0.296	1.81	0.74-4.42	0.192	1.46	0.86-2.47	0.159
HTN	0.92	0.43-1.95	0.821	0.74	0.30-1.87	0.528	0.89	0.51-1.57	0.693
Malignancy	0.12	0.03-0.49	0.003	0.64	0.20-2.05	0.449	0.30	0.13-0.69	0.005
Witnessed collapse	1.08	0.52-2.26	0.828	2.08	0.77-5.66	0.150	1.25	0.72-2.20	0.428
Epinephrine dose (mg)	0.76	0.67-0.87	< 0.001	0.86	0.74-0.99	0.036	0.81	0.74-0.89	< 0.001
CPR duration	1.00	0.98-1.03	0.781	0.95	0.91-0.99	0.020	0.99	0.96-1.01	0.288
DBP	1.02	1.00-1.04	0.134	1.00	0.97-1.03	0.993	1.01	0.99-1.03	0.224
SBP	1.00	0.99-1.01	0.815	1.01	0.99-1.02	0.399	1.00	0.99-1.01	0.910
pH value	6.06	0.66-55.61	0.111	1.77	0.11-28.23	0.686	3.31	0.60-18.28	0.170
Lactic acid	0.97	0.88-1.06	0.475	0.88	0.81-0.97	0.008	0.93	0.87-0.99	0.018
Troponin (ng/L)	0.99	0.98-1.01	0.288	0.99	0.99-1.00	0.204	0.99	0.99-1.00	0.074
ECMO	2.97	1.15-7.64	0.024	1.79	0.67-4.79	0.243	2.23	1.16-4.27	0.016

(b) With the automated method

	Derivation			Validation			Total		
	Odds Ratio	95% C.I.	P value	Odds Ratio	95% C.I.	P value	Odds Ratio	95% C.I.	P value
Automated_s > 1.202	3.70	1.96-7.01	< 0.001	3.65	1.64-8.13	0.002	3.61	2.22-5.86	< 0.001
Age	0.98	0.95-1.00	0.054	0.96	0.94-0.99	0.007	0.97	0.96-0.99	0.001
Male	1.46	0.72-2.96	0.293	1.88	0.76-4.63	0.171	1.53	0.90-2.62	0.119
HTN	0.88	0.41-1.90	0.738	0.73	0.29-1.83	0.503	0.87	0.50-1.54	0.637
Malignancy	0.10	0.03-0.41	0.001	0.83	0.26-2.66	0.754	0.28	0.12-0.66	0.004
Witnessed collapse	1.16	0.54-2.49	0.695	2.17	0.80-5.90	0.127	1.31	0.74-2.33	0.352
Epinephrine dose (mg)	0.78	0.69-0.89	< 0.001	0.89	0.77-1.02	0.091	0.83	0.76-0.91	< 0.001
CPR duration	1.00	0.97-1.03	0.883	0.95	0.91-0.99	0.020	0.98	0.96-1.01	0.145
DBP	1.01	0.99-1.04	0.227	1.00	0.97-1.03	0.990	1.01	0.99-1.02	0.344
SBP	1.00	0.99-1.01	0.693	1.01	0.99-1.02	0.464	1.00	0.99-1.01	0.981
pH value	3.28	0.32-33.10	0.315	1.30	0.08-22.25	0.856	2.10	0.36-12.21	0.411
Lactic acid	0.94	0.85-1.03	0.201	0.90	0.82-0.99	0.031	0.93	0.87-0.99	0.019
Troponin (ng/L)	0.99	0.98-1.01	0.334	0.99	0.98-1.00	0.216	0.99	0.99-1.00	0.088
ECMO	2.21	0.84-5.77	0.107	1.26	0.46-3.46	0.652	1.61	0.83-3.12	0.158

Abbreviations: C.I., confidence interval; HTN, hypertension; DBP, diastolic blood pressure; SBP, systolic blood pressure

Supplementary Table 3S: Comparison of survival to discharge between the manual method and automated method in the derivation and validation sets

	AUC (95% C.I.)	Sensitivity (95% C.I.)	Specificity (95% C.I.)	PPV (95% C.I.)	NPV (95% C.I.)	Cutoff value
**Derivation**						
Manual_b	0.71 (0.64-0.77)	0.69 (0.60-0.78)	0.68 (0.60-0.75)	0.60 (0.51-0.68)	0.76 (0.69-0.83)	1.200
Manual_s	0.69 (0.63-0.75)	0.62 (0.53-0.71)	0.69 (0.62-0.77)	0.58 (0.49-0.67)	0.73 (0.65-0.80)	1.244
Automated_b	0.72 (0.65-0.78)	**0.95** (0.91-0.99)	0.39 (0.32-0.47)	0.52 (0.45-0.59)	**0.92** (0.85-0.98)	1.177
Automated_s	**0.76** (0.70-0.82)	0.70 (0.62-0.79)	**0.72** (0.65-0.79)	**0.63** (0.54-0.72)	0.78 (0.71-0.85)	1.202
**Validation**						
Manual_b	0.65 (0.56-0.73)	0.61 (0.50-0.73)	0.55 (0.46-0.65)	0.50 (0.40-0.60)	0.66 (0.56-0.76)	1.200
Manual_s	0.65 (0.57-0.73)	0.61 (0.50-0.73)	0.60 (0.51-0.69)	0.53 (0.42-0.63)	0.68 (0.59-0.78)	1.244
Automated_b	0.68 (0.60-0.76)	**0.87** (0.79-0.94)	0.38 (0.29-0.47)	0.50 (0.42-0.59)	**0.80** (0.68-0.91)	1.177
Automated_s	**0.73** (0.65-0.80)	0.68 (0.57-0.79)	**0.69** (0.60-0.78)	**0.61** (0.51-0.72)	0.75 (0.66-0.83)	1.202

Abbreviations: C.I., confidence interval; PPV, positive predictive value; NPV, Negative predictive value

Supplementary Table 4S: DeLong's tests for comparing favorable neurological outcomes and survival to discharge between the manual and automated methods

	Manual_b	Manual_s	Automated_b	Automated_s
**Favorable neurological outcome**				
Manual_b	-	0.692	0.130	**0.001**
Manual_s	0.692	-	0.235	**0.002**
Automated_b	0.130	0.235	-	**< 0.001**
Automated_s	**0.001**	**0.002**	**< 0.001**	-
**Survival to discharge**				
Manual_b	-	0.731	0.414	**0.009**
Manual_s	0.731	-	0.297	**0.004**
Automated_b	0.414	0.297	-	**0.002**
Automated_s	**0.009**	**0.004**	**0.002**	-

Supplementary Table 5S: DeLong's tests for comparing favorable neurological outcomes and survival to discharge between multiple logistic regression models with manual and automated GWRs

	Without GWR	With Manual_b	With Manual_s	With Automated_b	With Automated_s
**Favorable neurological outcome**					
Without GWR	-	0.077	**0.027**	**0.014**	**0.003**
With Manual_b	0.077	-	0.335	0.238	**0.037**
With Manual_s	**0.027**	0.335	-	0.645	0.119
With Automated_b	**0.014**	0.238	0.645	-	0.090
With Automated_s	**0.003**	**0.037**	0.119	0.090	-
**Survival to discharge**					
Without GWR	-	**0.032**	**0.037**	**0.010**	**0.005**
With Manual_b	**0.032**	-	0.746	0.577	0.276
With Manual_s	**0.037**	0.746	-	0.432	0.168
With Automated_b	**0.010**	0.577	0.432	-	0.307
With Automated_s	**0.005**	0.276	0.168	0.307	-

Supplementary Table 6S: Favorable neurological outcome prediction performance for manual and automated GWRs at various cutoff values

	Sensitivity (95% C.I.)	Specificity (95% C.I.)	PPV (95% C.I.)	NPV (95% C.I.)
**Cutoff = 1.00**				
Manual_b	0.98 (0.96-1.00)	0.03 (0.01-0.05)	0.29 (0.25-0.33)	0.83 (0.60-1.00)
Manual_s	1.00 (1.00-1.00)	0.03 (0.02-0.06)	0.29 (0.25-0.34)	1.00 (1.00-1.00)
Automated_b	1.00 (1.00-1.00)	0.04 (0.02-0.06)	0.29 (0.25-0.34)	1.00 (1.00-1.00)
Automated_s	1.00 (1.00-1.00)	0.04 (0.02-0.06)	0.29 (0.25-0.34)	1.00 (1.00-1.00)
**Cutoff = 1.05**				
Manual_b	0.98 (0.96-1.00)	0.08 (0.05-0.11)	0.30 (0.26-0.35)	0.93 (0.81-1.00)
Manual_s	0.99 (0.97-1.00)	0.06 (0.04-0.09)	0.30 (0.25-0.34)	0.95 (0.83-1.00)
Automated_b	1.00 (1.00-1.00)	0.04 (0.02-0.07)	0.30 (0.25-0.34)	1.00 (1.00-1.00)
Automated_s	1.00 (1.00-1.00)	0.05 (0.03-0.07)	0.30 (0.25-0.34)	1.00 (1.00-1.00)
**Cutoff = 1.10**				
Manual_b	0.95 (0.91-0.99)	0.16 (0.12-0.20)	0.31 (0.27-0.36)	0.89 (0.81-0.97)
Manual_s	0.98 (0.96-1.00)	0.14 (0.10-0.18)	0.32 (0.27-0.36)	0.96 (0.89-1.00)
Automated_b	1.00 (1.00-1.00)	0.09 (0.06-0.12)	0.31 (0.26-0.35)	1.00 (1.00-1.00)
Automated_s	1.00 (1.00-1.00)	0.12 (0.09-0.16)	0.31 (0.27-0.36)	1.00 (1.00-1.00)
**Cutoff = 1.15**				
Manual_b	0.89 (0.83-0.94)	0.28 (0.23-0.33)	0.33 (0.28-0.38)	0.86 (0.79-0.93)
Manual_s	0.94 (0.89-0.98)	0.26 (0.21-0.31)	0.34 (0.29-0.39)	0.91 (0.85-0.97)
Automated_b	0.98 (0.96-1.00)	0.20 (0.15-0.24)	0.33 (0.28-0.38)	0.97 (0.92-1.00)
Automated_s	0.97 (0.93-0.99)	0.29 (0.24-0.34)	0.35 (0.30-0.40)	0.96 (0.91-0.99)
**Cutoff = 1.20**				
Manual_b	0.77 (0.70-0.84)	0.53 (0.48-0.59)	0.40 (0.34-0.46)	0.85 (0.80-0.90)
Manual_s	0.83 (0.76-0.89)	0.44 (0.39-0.50)	0.37 (0.31-0.43)	0.86 (0.81-0.92)
Automated_b	0.81 (0.74-0.87)	0.49 (0.43-0.54)	0.39 (0.33-0.45)	0.87 (0.81-0.91)
Automated_s	0.81 (0.74-0.88)	0.66 (0.61-0.71)	0.49 (0.42-0.56)	0.90 (0.86-0.93)
**Cutoff = 1.25**				
Manual_b	0.54 (0.46-0.63)	0.74 (0.70-0.79)	0.46 (0.38-0.54)	0.80 (0.76-0.85)
Manual_s	0.65 (0.56-0.73)	0.67 (0.62-0.73)	0.44 (0.37-0.51)	0.83 (0.78-0.87)
Automated_b	0.38 (0.29-0.47)	0.87 (0.83-0.91)	0.54 (0.44-0.64)	0.78 (0.73-0.82)
Automated_s	0.28 (0.20-0.36)	0.94 (0.92-0.97)	0.66 (0.53-0.79)	0.76 (0.72-0.81)
**Cutoff = 1.30**				
Manual_b	0.27 (0.19-0.35)	0.90 (0.87-0.93)	0.52 (0.40-0.65)	0.75 (0.71-0.80)
Manual_s	0.38 (0.29-0.46)	0.84 (0.80-0.88)	0.49 (0.39-0.59)	0.77 (0.73-0.81)
Automated_b	0.06 (0.02-0.10)	0.98 (0.96-0.99)	0.54 (0.25-0.81)	0.72 (0.68-0.76)
Automated_s	0.04 (0.01-0.08)	1.00 (0.99-1.00)	0.83 (0.50-1.00)	0.72 (0.68-0.76)

Abbreviations: C.I., confidence interval; PPV, positive predictive value; NPV, negative predictive value

Supplementary Table 7S: Compare AUC performance at different CT timing by hours

	Within 1 hr (95% C.I.) (N=92)	During 1-2 hr (95% C.I.) (N=253)	During 2-3 hr (95% C.I.) (N=66)	Over 3 hr (95% C.I.) (N=32)
Manual_b	**0.67** (0.55-0.77)	0.71 (0.62-0.78)	0.66 (0.50-0.82)	0.62 (0.39-0.83)
Manual_s	0.62 (0.51-0.74)	0.72 (0.65-0.79)	0.73 (0.59-0.86)	0.68 (0.47-0.87)
Automated_b	0.64 (0.51-0.75)	0.74 (0.68-0.81)	0.74 (0.59-0.86)	0.79 (0.59-0.94)
Automated_s	0.64 (0.52-0.75)	**0.81** (0.74-0.87)	**0.85** (0.74-0.94)	**0.84** (0.65-0.97)

Supplementary Table 8S: Compare AUC performance at different CT timing by median time of performing CT scans in the study

	Within 88 min (95% C.I.) (N=221)	Over 88 min (95% C.I.) (N=222)
Manual_b	0.68 (0.61-0.75)	0.69 (0.60-0.78)
Manual_s	0.67 (0.60-0.74)	0.73 (0.65-0.81)
Automated_b	0.72 (0.65-0.79)	0.74 (0.66-0.81)
Automated_s	**0.75** (0.68-0.81)	**0.83** (0.76-0.89)
